# Health-related quality of life in patients with neuroendocrine neoplasms: a two-wave longitudinal study

**DOI:** 10.1007/s40618-022-01872-w

**Published:** 2022-07-22

**Authors:** R. Modica, C. Scandurra, N. M. Maldonato, P. Dolce, G. G. Dipietrangelo, R. Centello, V. Di Vito, E. Giannetta, A. M. Isidori, A. Lenzi, A. Faggiano, A. Colao

**Affiliations:** 1 Department of Clinical Medicine and Surgery, University of Naples Federico II, Via Sergio Pansini 5, 80131 Naples, Italy; 2 Department of Neuroscience, Reproductive Sciences and Dentistry, University of Naples Federico II, Naples, Italy; 3 Department of Public Health, University of Naples Federico II, Naples, Italy; 4 Department of Experimental Medicine, «Sapienza» University of Rome, Rome, Italy; 5 Endocrinology Unit, Department of Clinical and Molecular Medicine, «Sapienza» University of Rome, Rome, Italy; 6 UNESCO Chair, Education for Health and Sustainable Development, Federico II University, Naples, Italy

**Keywords:** Neuroendocrine tumor, Quality of life, Resilience, Severity, Metastases, HRQoL

## Abstract

**Purpose:**

Scientific knowledge on health-related quality of life (HRQoL) in patients with neuroendocrine neoplasm (NEN) is still limited and longitudinal assessment of HRQoL over the time in NEN patients are scarce. The current study aimed to assess the role of clinical severity and heterogeneity of NEN, as well as resilience, in the HRQoL of NEN patients over the course of a year.

**Methods:**

39 consecutive NEN patients (25 men and 14 women) aged from 29 to 73 years participated in a longitudinal Italian multicentric study. The main outcome measure concerned the severity and heterogeneity of NEN, HRQoL, and resilience.

**Results:**

Over the course of a year, higher levels of the global health (GH) were associated to the absence of distant metastases, while the presence of metastases with higher levels of fatigue, diarrhea, and financial difficulties. Higher levels of resilience are still associated with better GH and lower levels of fatigue, diarrhea, and financial difficulties, but no longer with constipation. Furthermore, patients with gastroenteropancreatic NEN still have higher scores on constipation, but not on GH, fatigue, diarrhea, and financial difficulties. Patients with hereditary NEN continue to have greater GH than those with a sporadic NEN and lower fatigue, diarrhea, and financial difficulties.

**Conclusion:**

These findings showed that the effects of severity and clinical heterogeneity of the NEN on HRQoL may change over time. This evidence should lead clinicians to monitor the HRQoL of NEN patients throughout the course of the disease and psychologists to implement evidence-based resilience interventions.

## Introduction

Neuroendocrine neoplasms (NEN) are relatively rare tumors with peculiar biological characteristics and variable clinical presentation. They mostly arise in the gastroenteropancreatic (GEP) and pulmonary systems but can affect any organ and tissue in the body where cells of the diffused neuroendocrine system are found. NEN are mainly sporadic but can be part of hereditary syndromes as multiple endocrine neoplasia (MEN 1), and in the latter case, they occur earlier in age and are often small and multiple [1].

The difference in clinical behavior between sporadic and genetic NEN may have an impact on disease perception because the association of endocrine and nonendocrine disorders as in MEN1 could increase the disease-related worries, while sporadic NEN may perceive a greater chance to recovery, although often diagnosed in more advanced stages [2]. NEN incidence is steadily increasing from 1.09 per 100,000 in 1973 to 6.98 per 100,000 in 2012 regardless of site, stage, and grade [3]. Furthermore, NEN are often associated with metastases mainly to the liver, already at diagnosis (40–76%), despite a usually slow growing behavior [4]. Many treatments have become available in recent years, including systemic therapies (e.g., somatostatin analogs (SSA)), targeted therapies (e.g., everolimus and sunitinib), liver directed therapies or external beam radiotherapy, peptide receptor radionuclide therapy (PRRT), and chemotherapy. Consequently, variable therapeutic sequences have been proposed, but all therapies may have toxicities, even cumulative [5, 6]. These advances may have an impact on overall survival (OS), which is improving with time, as the median OS is 9.3 years [3]. On the other hand, a longer survival together with the use of different therapeutic strategies imply a higher impact of quality of life in the management of these patients.

Such a complex clinical picture may support the concept of NEN as a chronic illness, thus making crucial the assessment of the health-related quality of life (HRQoL) over time [7, 8]. The assessment of HRQoL in patients with NEN is gaining increasing interest in recent years, as demonstrated by its inclusion as an endpoint also in clinical trials, but our knowledge on HRQoL in this group of cancers is still scarce, in particular whether longitudinal study designs are considered [9–12]. A recent meta-analysis of 64 studies including 28,423 participants analyzed HRQoL in long-term cancer survivors and reported that HRQoL is significantly impacted 2–26 years after cancer diagnosis. Furthermore, since physical and mental health have a major role in HRQoL, the impact of potential moderators, such as resilience, is of utmost importance [8]. Previous studies suggested that patients with NEN tend to perceive their HRQoL as relatively good, even if both physical and psychosocial complaints are often reported (e.g., fatigue, sleep problems, poor emotional, mental, physical, and social functioning) [13, 14]. In a cross-sectional survey conducted by the authors of the current work and aimed at assessing the role of clinical severity and heterogeneity of NEN in relation to HRQoL, as well as resilience as a moderator between clinical severity and HRQoL, it was found that: (1) having metastases and undergoing a greater number of therapies affected the HRQoL of patients with NEN; (2) patients with NEN in districts other than the GEP system and in follow-up had a better HRQoL than their counterparts; (3) sporadic NEN were less impacting than hereditary NEN; and (4) resilience buffered the effects of severity on some symptoms, including constipation, diarrhea, and financial problems [2].

Based on our previous study [2], the current two-wave longitudinal study aimed to assess whether the clinical severity and heterogeneity of NEN continue to affect the HRQoL of patients with NEN over the course of a year. Furthermore, this study aimed to assess whether even resilience is able to protect patients against the hypothesized negative effects of NEN on HRQoL over the course of a year.

## Materials and methods

### Procedures and patients

The data analyzed in the current study represent the second wave of collection of the Italian project “A multicentric clinical study on the quality of life in patients with neuroendocrine neoplasms,” whose preliminary assessment was published in Scandurra et al. [2].

Patients were recruited in two Italian clinical centers: the Unit of Neuroendocrine Neoplasms of the University of Naples Federico II and the NETTARE Unit of “Sapienza” University of Rome. The first assessment started in September 2019 and ended in February 2020, while the second assessment started 1 year after the end of the first wave (i.e., from February 2021 to April 2021). Patients were managed mainly by endocrinologists, but in the context of a multidisciplinary tumor board, given the heterogeneity of NEN.

Patients were eligible if they: (1) were aged between 18 and 75 years; (2) had a histologically confirmed NEN diagnosis; (3) were able to autonomously understand and sign the informed consent; and (4) were able to complete the questionnaire independently. Each patient provided an informed written consent to participate in the study and to publish results in aggregate and anonymous form. Patients with other secondary primary malignant neoplasms were excluded.

Among 99 patients who participated in the first wave of assessment, 39 accepted to complete the second wave assessment. The remaining 60 patients did not answer to questionnaires for different reasons as follows: 10 patients died on follow-up, 18 were lost on follow-up and 32 refused to respond. The included patients ranged in age from 29 to 73 years (*M* = 54.87; SD = 12.72). Twenty-five were males and 14 females. Furthermore, 86.1% of the sample had an educational level ≤ high school, while 13.9% an ≥ university college.

The study was conducted in accordance with the Declaration of Helsinki and the EU General Data Protection Regulation and was approved by the Ethical Committee of the University of Naples Federico II (project identification code: 156/2019; date of approval: 25th July 2019).

### Measures

#### Severity and clinical heterogeneity of the NEN

Severity of the NEN was assessed through two dimensions: (1) presence vs. absence of metastasis; and (2) number of therapies experienced (from 0 = no therapies to 2 = two lines of therapies).

Clinical heterogeneity of the NEN was assessed by considering 4 medical outcomes collected through a clinician-report, as follows: (1) site of the NEN (GEP vs. non-GEP); (2) type of current therapies (from less to more severe therapy, i.e.: 1 = follow-up without therapy but with MEN1; 2 = SSA; and 3 = other therapies); (3) nature of the NEN (sporadic vs. hereditary); and 4) surgery vs. no-surgery.

### Health-related quality of life

HRQoL was assessed through the Italian versions of the EORTC Quality of Life Questionnaire-C30 (EORTC QLQ-C30, version 3.0) and the EORTC Quality of Life Questionnaire Neuroendocrine Carcinoid Module (EORTC QLQ-GINET21). In the current study, we reported data only about the EORTC QLQ-C30 for simplicity. EORTC QLQ-C30 is a widely used measure of HRQoL in cancer patients and assessed the levels of 5 functional status scales (i.e., physical, role, emotional, cognitive, and social functioning), 9 symptoms (fatigue, nausea and vomiting, pain, dyspnea, insomnia, appetite loss, constipation, diarrhea, and financial difficulties), and global health (GH) [15]. Higher scores on the functional scales and GH indicate higher level of HRQoL, while higher scores on the symptom scales reflect lower levels of HRQoL. In the current study, only HRQoL variables that resulted statistically significant in our previous study [2] have been considered, as follows: GH, fatigue, constipation, diarrhea, and financial difficulties.

### Resilience

Resilience was assessed through the Resilience Scale for Adults (RSA) [16], a 33-item measure evaluating the ability to exhibit resourcefulness in response to adverse and stressful events. The measure uses a 7-point semantic differential scale format, with a positive and a negative attribute at each side of the continuum and with higher scores indicating greater resilience. The α coefficient for the current sample was 0.85 in participants of the first wave of measurement and 0.80 in those of the second wave.

### Statistical analyses

Means and standard deviations were used to summarize quantitative variables while frequencies (percentages) to summarize categorical variables.

Differences on HRQoL and resilience between the first and the second wave were assessed using mixed-effects models using all the available data at each time point, considering intercept for subjects as random effects and time as a fixed-effect parameter, and, thus, testing for statistical differences from the first and the second wave.

Relations over the course of a year between HRQoL and severity of NEN (i.e., presence vs. absence of metastasis and number of therapies experienced) and clinical heterogeneity (site of the NEN, type of current therapies, tumor biology, and surgery vs. no-surgery) were also analyzed using mixed-effects models by considering intercept for subjects as random effects and each clinical variable as a fixed-effect parameter. Mixed-effects models were run separately for each HRQoL score and each clinical variable. Regarding the severity of NEN, the analyses were adjusted for resilience.

All statistical analyses were conducted using the R statistical software. Mixed-effects models were fitted using the lmer function in the lme4 R package [17]. The level of significance for all the statistical tests was set at 0.05.

## Results

### Clinical status of participants

Considering the 39 patients who participated in the second wave of measurement, the average latency from NEN diagnosis was 7.33 years (SD = 7.04). Twenty-eight (71.8%) patients had a NEN in the gastrointestinal tract as follows: pancreas (*n* = 18; 46.1%), small intestine (*n* = 6; 15.4%), stomach (*n* = 4; 10.2%). The remaining 11 (28.2%) had a NEN in other sites, as follows: thyroid (*n* = 8; 20.5%), lungs (*n* = 1; 2.6%), oropharynx (*n* = 1; 2.6%), and adrenals (*n* = 1; 2.6%).

Most patients (*n* = 23; 59%) were undergoing SSA, 9 (23.1) were in follow-up without any therapy, and 7(17.9%) were undergoing other types of therapy (e.g., peptide receptor radionuclide therapy, targeted therapy, chemotherapy, etc.). Seven (17.9%) patients had not undergone any therapy, 24(61.5%) had undergone one line of therapy, and 8(20.5%) had undergone two lines of therapy. Twenty-nine patients (74.4%) had a sporadic NEN, 20(51.3%) a metastatic tumor, and only 1(2.6%) underwent surgery in the past.

### Differences between participants of the two waves

The only difference we found over the course of a year concerned resilience. Specifically, resilience increased on the second wave (*M* = 21.87, SD = 2.98 vs. *M* = 23.05, SD = 3.65, *p* = 0.030). All other variables (i.e., GH, constipation, fatigue, diarrhea, and financial difficulties) did not differ from the first wave to the second wave.

### Associations between HRQoL and Severity of NEN controlling for Resilience

The results for the mixed effect model of HRQoL on the presence vs. absence of metastases and resilience are presented in Table 1. Patients without metastases continue to have a higher GH score than their counterparts, while those with metastases continue to have higher fatigue, diarrhea, and financial difficulties than patients without metastases. Unlike the first wave of measurement, the presence of metastases is no longer significantly associated with constipation. Similarly, higher levels of resilience result yet associated with a better GH score and lower levels of fatigue, diarrhea, and financial difficulties, but no longer with constipation.

**Table 1:** Mixed effect model of HRQoL on the presence vs. absence of metastases and resilience over the course of a year

HRQoL	Metastasis		Resilience
	*b*		*b*
Global health score	− 9.45*		1.89***
Fatigue	12.3*		− 1.85**
Constipation	− 1.09		− 0.77
Diarrhea	14.7**		− 1.83*
Financial difficulties	14.7**		− 1.83*

HRQoL health-related quality of life, *b* standardized regression coefficient

*p < 0.05, **p < 0.01, ***p < 0.001

The results for the mixed effect model of HRQoL on the number of current therapies and resilience are reported in Table 2. A greater number of therapies is still associated with a lower GH score and higher levels of diarrhea and financial difficulties, but no longer with fatigue and constipation. Again, higher levels of resilience are still associated with a better GH score and lower levels of fatigue, diarrhea, and financial difficulties, but no longer with constipation.

**Table 2:** Mixed effect model of HRQoL on the number of current therapies and resilience over the course of a year

HRQoL	Number of current therapies	Resilience
	*b*	*b*
Global health score	− 6.97*	1.96***
Fatigue	4.41	− 1.82**
Constipation	− 0.35	− 0.80
Diarrhea	10.3*	− 1.78*
Financial difficulties	10.3*	− 1.78*

HRQoL health-related quality of life, b standardized regression coefficient

*p < 0.05, **p < 0.01, ***p < 0.001

### Clinical heterogeneity and HRQoL

The results for the mixed effect model of HRQoL-related differences concerning the site of NEN (GEP vs. non-GEP), current therapies (follow-up vs. SSA vs. other), tumor biology (hereditary vs. sporadic), and surgery (yes vs. no) are presented in Table 3

**Table 3:** HRQoL according to the site of the NEN, current therapies, tumor biology, and surgery over the course of a year

	Site of the NEN	Current therapies		Tumor biology	Surgery
	GEP *b*	SSA *b*	Other *b*	Hereditary *b*	Yes *b*
Global health score	− 3.01	− 2.28	− 1.66	13.5**	− 0.97
Fatigue	6.33	− 0.57	5.87	− 18.6***	7.68
Constipation	11.52*	− 5.59	− 5.93	3.3	− 0.93
Diarrhea	− 4.62	13.4*	26.2**	− 12.2*	4.33
Financial difficulties	− 4.62	13.4*	26.2**	− 12.2*	4.33

Patients with a GEP-NEN continue to have higher scores than patients with a non-GEP NEN on constipation, but not on all other HRQoL dimensions. Furthermore, patients in follow-up continue to present lower levels than those undergoing SSA or other therapies in diarrhea and financial difficulties.

Patients with hereditary NEN continue to have greater GH than those with a sporadic NEN and lower fatigue, diarrhea, and financial difficulties. Instead, the difference detected by Scandurra et al. [2] on constipation between patients who underwent surgery and those who did not undergo any surgery is no longer significant [2].

## Discussion

The current study has assessed the relationships between clinical severity, heterogeneity of NEN, and resilience with the HRQoL of a sample of Italian patients with NEN in two waves of measurement. In our previous study we found that the presence of metastasis and a higher number of therapies negatively affected the global health and some physical symptoms in NEN, and that resilience positively influenced the impact of metastases on constipation and of the multiple therapies on diarrhea and financial problems [2]. In this new assessment after 1 year, only some of the first-wave findings have been confirmed, shedding light on the importance of assessing the potential changes of psychological and medical variables over the course of the time.

Results regarding the associations between severity of NEN, HRQoL, and resilience generally confirmed that both the presence of metastases and a greater number of therapies continue to negatively affect the HRQoL of patients with NEN over the course of a year. The prevalence of metastases is high in NET even at diagnosis and the prolonged course of disease often requires different therapeutic sequences; thus, it is quite expected that these factors negatively influence the HRQoL in this later evaluation as already demonstrated in the previous study [2, 4].

Another interesting finding is that resilience still protects NEN patients from the negative effects of tumor on HRQoL. Resilience in cancer care can be defined as a dynamic process of facing adversity related to cancer that can be modulated through interventions [18, 19]; thus, the confirmation of its protective role during the disease poses the challenge to identify interventions that may reinforce it.

The positive role of resilience has already been demonstrated in patients with breast cancer with a positive association with GH/QoL score and functional scales and a negative association with physical symptoms [20]. Even in patients treated for cancer of the head and neck, resilience was associated with overall HRQoL and socioemotional functioning [21]. More recently, resilience was analyzed in patients who survived at least 5 years after lung cancer surgery and it changed over time across 3 stages that included initial stress, adaptation to disease, and personal growth [22]. These stages could be more difficult to define in NEN patients as they often have a chronic and slow growing disease, but the concept that resilience is an evolving process must be taken into consideration when finding protective factors.

However, with respect to the first wave of measurement, neither the presence of metastases nor the high number of therapies continue to affect the symptom of constipation over the course of a year. Gastrointestinal symptoms as constipation and diarrhea may be common in GEP-NEN and could be affected by the kind of therapies rather than their number [5]. In the current study, the high number of therapies no longer affects the fatigue. Fatigue is a common and non-specific symptom in cancer patients that may vary during the survivorship and is due to complex multifactorial processes [23]. In a study that investigated the impact of treatment on HRQoL in 663 NEN patients, fatigue was more prevalent in patients with recurrent NEN than in those with no current NET, thus fatigue should be more related to the disease than to therapies [24].

Results regarding the associations between clinical heterogeneity and HRQoL did not confirm the relationships of both the site of the NEN and the current therapies with HRQoL as, among the HRQoL variables considered, only constipation for the site of NEN and diarrhea and financial difficulties continue to be significant. Similarly, having undergo surgery no longer affects the symptom of constipation, but this symptom that may be easily managed with supportive therapy during disease. The evaluation of HRQoL-related to therapies remains questioned and in a comment regarding therapy with everolimus it has also been speculated that no good methods are available to assess small differences in HRQoL between treatments for NEN, thus a long-term assessment of HRQoL in these patients has been advocated [25]. The only dimension which resulted to be relatively stable is the difference between hereditary and sporadic NEN, as having a hereditary NEN continue to affect the GH more than having a sporadic NEN but, at the same time, these patients have shown lower symptomatic levels (i.e., fatigue and diarrhea) and financial troubles than their sporadic counterpart. As already described in our first evaluation, we can suppose that the worse GH of hereditary NEN patients could be related to the association of several endocrine and nonendocrine diseases that may increase their disease-related worries. Nevertheless, hereditary NEN patients show less clinical symptoms than patients with sporadic NEN and it may be related to the earlier diagnosis, often due to genetic screening. Furthermore, hereditary NEN are often diagnosed in earlier stage compared to sporadic counterpart and are asymptomatic or with symptoms that are manageable with follow-up or well tolerated therapies as SSA [26]. Finally, although we included 10 patients with hereditary NEN and 29 with sporadic NEN and it may have influenced our results, we believe that this discrepancy reflects the epidemiological difference [1].

Results of the current study must be read considering important limitations. First, not all the patients who took part at the first wave of the study answered also at the second wave, making the sample size quite small. Additionally, this prevented the possibility to assess potential differences in severity and heterogeneity, as well as their potential impact on HRQoL, over the course of time. However, the aim of this study was to assess whether the clinical severity, heterogeneity of NEN, and resilience still have an impact on HRQoL over the course of a year, rather than assessing means differences of the variables. Second, because of the small sample size, it was not possible to perform some subgroup analyses, including age and site of primary, as dividing the data into subgroups would have affected the validity and precision of the statistical analysis. Third, the latency from diagnosis was 7.33 years in the current study and almost the same in the original study (i.e., 7.26 years), suggesting that the missing population must have had a longer latency to diagnosis on average, thus representing a significant bias that may have affected our results. Fourth, the difference in resilience scores in the follow-up study is possibly indicative of a bias due to drop-out of patients with lower resilience scores. Fifth, unlike the original study, the data in the current study were collected during the Covid-19 outbreak, whose restrictive measures imposed by governments to contain the spread of the SARS-CoV-2 (e.g., lockdowns, social distancing, quarantine, etc.) impacted HRQoL and resilience in the general population as well as cancer patients [27]. Thus, although the Italian NEN network was able to provide continuity of care without discontinuing antitumor treatment for most patients [28], our results are likely to be in part affected by the effects that the restrictions and the pandemic in general have had on people’s health and wellbeing. Given these limitations, it is important to note that patients lost to follow-up may have had different latency to diagnosis and different resilience scores, representing a potential bias that may have affected results. The heterogeneity of NEN would certainly have required a larger sample size to obtain reliable data, but unfortunately the relative rarity of the disease and its long natural history with different therapeutic options make it difficult to study a large number of patients with similar characteristics. For example, patients’ characteristics that could potentially affect both resilience and HRQoL may be age, gender, body max index, and second primary malignancies. Indeed, a relatively high prevalence of second primary malignancies has been recently demonstrated, suggesting a possible neoplastic susceptibility, although not affecting the overall survival [29]. Furthermore, long-term follow-up and reevaluation of this cohort of patients will shed more light on HRQoL, while the presence of NEN of different site of origin may depict a representative example of the heterogeneity of these tumors in the real-world condition of daily clinical routine. However, thanks to the Italian Association for Neuroendocrine Tumors (It.a.net), which supported this study, we plan to extend our research on HRQoL in NEN to other Italian NET centers in the near future to increase the sample size and allow comparative analyses.

## Conclusions

The findings of this study deepen our understanding on the HRQoL of patients with NEN. As shown, the effects of severity and clinical heterogeneity of the NEN on HRQoL may change over time. This evidence should lead clinicians to monitor the HRQoL of their patients throughout the course of the disease. At the same time, the crucial role of resilience should lead psychologists to implement evidence-based resilience interventions for patients with NEN.

## Funding sources

Partial financial support was received from the Italian Ministry of Education, University and Research (PRIN 2017Z3N3YC). The authors have no competing interests to declare that are relevant to the content of this article.

## Data Availability

The datasets generated during and/or analyzed during the current study are not publicly available but are available from the corresponding author on reasonable request.

## References

[1] Frilling A, Åkerström G, Falconi M, Pavel M, Ramos J, Kidd M, Modlin IM (2012). Neuroendocrine tumor disease: an evolving landscape. Endocr Relat Cancer.

[2] Scandurra C, Modica R, Maldonato NM, Dolce P, Dipietrangelo GG, Centello R, di Vito V, Bottiglieri F, de Cicco F, Giannetta E, Isidori AM, Lenzi A, Muzii B, Faggiano A, Colao A (2021). Quality of life in patients with neuroendocrine neoplasms: the role of severity, clinical heterogeneity, and resilience. J Clin Endocrinol Metab.

[3] Dasari A, Shen C, Halperin D, Zhao B, Zhou S, Xu Y, Shih T, Yao JC (2017). Trends in the incidence, prevalence, and survival outcomes in patients with neuroendocrine tumors in the United States. JAMA Oncol.

[4] Das S, Dasari A (2021). Epidemiology, incidence, and prevalence of neuroendocrine neoplasms: are there global differences?. Curr Oncol Rep.

[5] Faggiano A, lo Calzo F, Pizza G, Modica R, Colao A,  (2017). The safety of available treatments options for neuroendocrine tumors. Expert Opin Drug Saf.

[6] Faggiano A, di Maio S, Mocerino C, Ottaviano M, de Divitiis C, Guarnotta V, Dolce P, Modica R, Puliafito I, Tozzi L, di Sarno A, Leo S, Riccardi F, Palmieri G, Tafuto S, Bianco A, Badalamenti G, Colao A (2019). Therapeutic sequences in patients with grade 1–2 neuroendocrine tumors (NET): an observational multicenter study from the ELIOS group. Endocrine.

[7] Beesley VL, Burge M, Dumbrava M, Callum J, Neale RE, Wyld DK (2018). Perceptions of care and patient-reported outcomes in people living with neuroendocrine tumours. Supp Care Cancer.

[8] Firkins J, Hansen L, Driessnack M, Dieckmann N (2020). Quality of life in “chronic” cancer survivors: a meta-analysis. J Cancer Surviv.

[9] Jiménez-Fonseca P, Carmona-Bayonas A, Martín-Pérez E, Crespo G, Serrano R, Llanos M, Villabona C, García-Carbonero R, Aller J, Capdevila J, Grande E (2015). Health-related quality of life in well-differentiated metastatic gastroenteropancreatic neuroendocrine tumors. Cancer Metastasis Rev.

[10] Haugland T, Devon HA (2019). Symptoms, psychosocial factors, and health-related quality of life in patients with neuroendocrine tumors: An integrative review. Cancer Nurs.

[11] White BE, Druce MR, Grozinsky-Glasberg S, Srirajaskanthan R, Gamper EM, Gray D, Mujica-Mota R, Ramage JK (2020). Health-related quality of life in neuroendocrine neoplasia: a critical review. Endocr Relat Cancer.

[12] Martini C, Gamper EM, Wintner L, Nilica B, Sperner-Unterweger B, Holzner B, Virgolini I (2016). Systematic review reveals lack of quality in reporting health-related quality of life in patients with gastroenteropancreatic neuroendocrine tumours. Health Qual Life Outcomes.

[13] Hallet J, Davis LE, Mahar AL, Isenberg-Grzeda E, Bubis LD, Myrehaug S, Zhao H, Beyfuss K, Moody L, Law CHL, Coburn NG (2019). Symptom burden at the end of life for neuroendocrine tumors: an analysis of 2579 prospectively collected patient-reported outcomes. Ann Surg Oncol.

[14] Martini C, Buxbaum S, Rodrigues M, Nilica B, Scarpa L, Holzner B, Virgolini I, Gamper EM (2018). Quality of life in patients with metastatic gastroenteropancreatic neuroendocrine tumors receiving peptide receptor radionuclide therapy: information from a monitoring program in clinical routine. J Nucl Med.

[15] Aaronson NK, Ahmedzai S, Bergman B, Bullinger M, Cull A, Duez NJ, Filiberti A, Flechtner H, Fleishman SB, Haes JCJMD, Kaasa S, Klee M, Osoba D, Razavi D, Rofe PB, Schraub S, Sneeuw K, Sullivan M, Takeda F (1993). The European organization for research and treatment of cancer QLQ-C30: a quality-of-life instrument for use in international clinical trials in oncology. J Natl Cancer Inst.

[16] Hjemdal O, Friborg O, Braun S, Kempenaers C, Linkowski P, Fossion P (2011). The resilience scale for adults: construct validity and measurement in a Belgian sample. Int J Test.

[17] Bates D, Mächler M, Bolker BM, Walker SC (2015). Fitting linear mixed-effects models using lme4. J Stat Softw.

[18] Eicher M, Matzka M, Dubey C, White K (2015). Resilience in adult cancer care: an integrative literature review. Oncol Nurs Forum.

[19] Matzka M, Mayer H, Köck-Hódi S, Moses-Passini C, Dubey C, Jahn P, Schneeweiss S, Eicher M (2016). Relationship between resilience, psychological distress and physical activity in cancer patients: a cross-sectional observation study. PLoS ONE.

[20] Ristevska-Dimitrovska G, Filov I, Rajchanovska D, Stefanovski P, Dejanova B (2015). Resilience and quality of life in breast cancer patients. Open Access Maced J Med Sci.

[21] Clarke G, Asiedu YA, Herd K, Sharma S (2019). Exploring the relation between patients’ resilience and quality of life after treatment for cancer of the head and neck. Br J Oral Maxillofac Surg.

[22] Li X, Chen S, Zhang J, Li L, Li Y, Ye M (2021). Resilience process and its protective factors in long-term survivors after lung cancer surgery: a qualitative study. Supp Care Cancer.

[23] Thong MSY, van Noorden CJF, Steindorf K, Arndt V (2020). Cancer-related fatigue: Causes and current treatment options. Curr Treat Options Oncol.

[24] Pearman TP, Beaumont JL, Cella D, Neary MP, Yao J (2016). Health-related quality of life in patients with neuroendocrine tumors: an investigation of treatment type, disease status, and symptom burden. Supp Care Cancer.

[25] Walter T (2017). Maintaining quality of life for patients with neuroendocrine tumours. Lancet Oncol.

[26] Faggiano A, Modica R, Calzo F, Camera L, Napolitano V, Altieri B, de Cicco F, Bottiglieri F, Sesti F, Badalamenti G, Isidori AM, Colao A (2019). Lanreotide therapy vs active surveillance in MEN1-related pancreatic neuroendocrine tumors 2 centimeters. J Clin Endocrinol Metab.

[27] Rossi R, Jannini TB, Socci V, Pacitti F, Lorenzo G (2021). Stressful life events and resilience during the COVID-19 lockdown measures in Italy: Association with mental health outcomes and age. Front Psychiatry.

[28] Panzuto F, Maccauro M, Campana D, Faggiano A, Massironi S, Pusceddu S, Spada F, Ferone D, Modica R, Grana CM, Ferolla P, Rinzivillo M, Badalamenti G, Zatelli MC, Gelsomino F, de Carlo E, Bartolomei M, Brizzi MP, Cingarlini S, Versari A, Fanciulli G, Arvat E, Merola E, Cives M, Tafuto S, Baldari S, Falconi M (2021). Impact of the SARS-CoV2 pandemic dissemination on the management of neuroendocrine neoplasia in Italy: a report from the Italian Association for Neuroendocrine Tumors (Itanet). J Endocrinol Investig.

[29] Massironi S, Campana D, Pusceddu S, Albertelli M, Faggiano A, Panzuto F, Smiroldo V, Andreasi V, Rossi RE, Maggio I, Torchio M, Dotto A, Modica R, Rinzivillo M, Carnaghi C, Partelli S, Fanetti I, Lamberti G, Corti F, Ferone D, Colao A, Annibale B, Invernizzi P, Falconi M, ItaNet (Italian Association for Neuroendocrine Tumours) (2021). Study Group. Second primary neoplasms in patients with lung and gastroenteropancreatic neuroendocrine neoplasms: Data from a retrospective multi-centric study. Dig Liver Dis.

